# The Contribution of Major Histocompatibility Complex Class II Genes to an Association with Autoimmune Diseases

**DOI:** 10.32607/20758251-2019-11-4-4-12

**Published:** 2019

**Authors:** M. Yu. Zakharova, T. A. Belyanina, A. V. Sokolov, I. S. Kiselev, A. E. Mamedov

**Affiliations:** Shemyakin-Ovchinnikov Institute of Bioorganic Chemistry, Russian Academy of Sciences, Moscow, 117997 Russia; Pirogov Russian National Research Medical University, Moscow, 117997 Russia; I.M. Sechenov First Moscow State Medical University, Moscow, 119991 Russia

**Keywords:** antigen presentation, autoimmune diseases, human leukocyte antigen, major histocompatibility complex, multiple sclerosis, rheumatoid arthritis, type 1 diabetes

## Abstract

Genetic studies of patients with autoimmune diseases have shown that one of the most important roles in the developing of these diseases is played by a cluster of genes of the major histocompatibility complex (MHC), as compared with other genome areas. Information on the specific contribution of MHC alleles, mostly MHC class II ones, to the genetic predisposition to autoimmune diseases is crucial for understanding their pathogenesis. This review dwells on the most relevant aspects of this problem: namely, the correlation between carriage of certain MHC II alleles and an increased (positively associated allele) or reduced (negatively associated allele) probability of developing the most common autoimmune diseases, such as type 1 diabetes, rheumatoid arthritis, multiple sclerosis, systemic lupus erythematosus, autoimmune thyroiditis, etc. The most universal haplotypes, DR3-DQ2 and DR4-DQ8, are positively associated with many of these diseases, while the universal allele HLA-DRB1*0701 is protective.

## INTRODUCTION

**Fig. 1:**
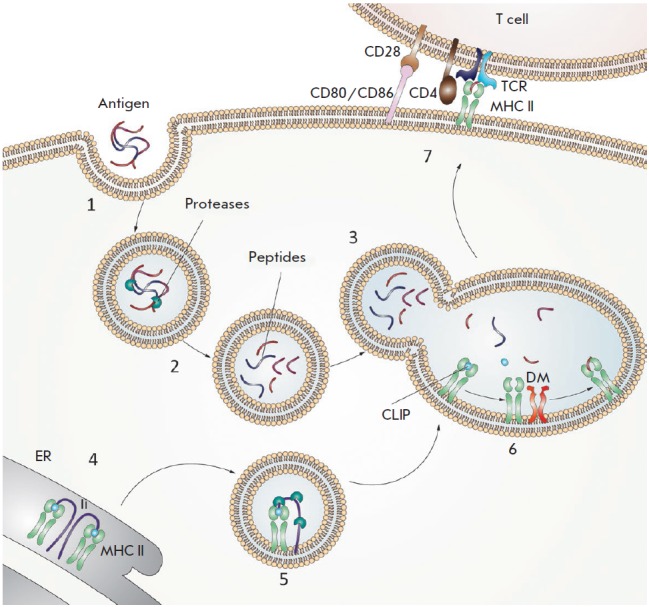
Diagram showing antigen presentation by MHC II molecules. (1) An antigen enters intracellular vesicles. (2) Acidification of vesicles activates proteases that hydrolyze the antigen into peptide fragments. (3) Vesicles containing the peptide fragments merge with vesicles containing MHC II molecules (green). (4) The invariant chain (Ii) (violet) binds to the newly synthesized MHC II molecules and partially occupies the peptide-binding groove. (5) The invariant chain undergoes proteolytic degradation; as a result, the CLIP peptide (blue) remains bound in the groove. (6) DM (orange) binds to the MHC II molecules and catalyzes the peptide exchange. (7) The MHC II molecules, loaded with an antigenic peptide (red), are transported to the cell surface where they can be recognized by a CD4 + T cell receptor TCR (cyan blue). The CD4 co-receptor molecule (brown) present on T cells also binds to the MHC II molecules. For T-cell activation to occur, the CD80 or CD86 co-stimulating molecules (pink) expressed on the antigen-presenting cell must bind to the CD28 co-stimulating molecule (beige) expressed on the T cells

The major histocompatibility complex (MHC), or human leukocyte antigen (HLA), contains several gene clusters that encode surface heterodimeric proteins, which are anchored to the plasma membrane and are responsible for antigen presentation to T cells, a stage that is followed by the development of an adaptive immune response. MHC proteins are subdivided into class I, class II, and class III (the complement system) [1]. MHC class I proteins occur in almost all cell types and are involved in the presentation of self-antigens fragments, which trigger the CD8^+^ T cell-mediated immune response. MHC class I molecules are found on the surface of professional antigen-presenting cells (APCs) and mostly present fragments of foreign antigens (bacterial, viral, etc.) captured by APCs. The MHC II–peptide complex interacts with CD4^+^ T cells (*Fig. 1*).

MHC proteins are heterodimers that consist of two chains: the long α chain containing a transmembrane domain and a short universal β2-microglobulin chain (for MHC I), or long α and β chains carrying extracellular, transmembrane and short cytoplasmic domains (for MHC II). The peptide-binding groove is an essential structural element of MHC, because its structure is responsible for peptide binding and further triggering of the immune response. HLA molecules need to be highly polymorphic to ensure presentation of a large number of variable peptides.

**Fig. 2:**
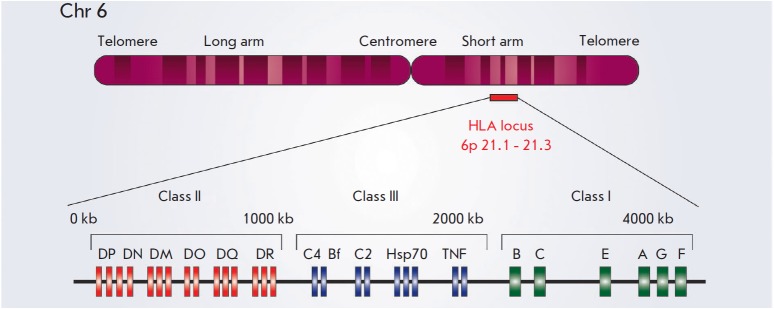
Schematic representation of the HLA locus on human chromosome 6. The HLA region is located on the short arm of chromosome 6 from 6p21.1 to p21.3 and is shown with a red stripe. The length of class II (red), class III (blue), and class I (green) genes (from the centromeric to the telomeric end) is shown. The class II region includes genes for the α and β chains of the MHC class II molecules HLA-DR, HLA-DP and HLA-DQ. In addition, the genes encoding the DMα and DMβ chains, as well as the genes encoding the α and β chains of the DO molecule (DOα and DOβ, respectively), are also located in the MHC class II region

MHC genes are located on chromosome 6 (except for the gene for the light chain of MHC I (β2- microglobulin), which resides on chromosome 15) and form extensive clusters (*Fig. 2*). Class I genes include *HLA-A, HLA-B*, and *HLA-C*, which encode the α chains of the heterodimer. MHC class II molecules are mainly encoded by the genes of the HLA-DR, HLA-DQ, and HLA-DP loci; each of them includes an α and β chain gene (e.g., the *DRA1 *gene coding for the α chain and the *DRB1*, *DRB3*, *DRB4*, and *DRB5 *genes coding for β chains in the HLA-DR locus). This nomenclature has evolved through historical sequences of discovery of HLAs: they were named using Roman numerals and English alphabet letters as they were progressively discovered.

The MHC locus is the most polymorphic in the human genome [2]. It is responsible for the existence of a vast diversity of MHC protein forms. To classify the products of these genes’ expression, MHC molecules were subdivided into groups, in accordance with their serotypes (e.g., the HLA-DR1 serogroup). The advances in molecular genetic methods have made it possible to refine the nomenclature and identify the groups of *HLA *alleles that correspond to serogroups of their protein products (DRA*01 + DRB1*01, respectively) and subsequently even the specific gene alleles (DRA*01 + DRB1*0101, *0102 or *0103, respectively) [3]. The *HLA-B *gene is the most polymorphic MHC class I gene (with 1,077 alleles reported); the *HLA-DRB1 *gene, with 669 alleles, is the most polymorphic among MHC class II genes [4, 5]. Extensive linkage disequilibrium (LD) regions (up to 500 kb) were found within the *HLA *genomic region [6]. These extensive inherited gene clusters complicate the identification of specific disease-associated alleles, since they often cannot be differentiated from the full inherited haplotype.

To date, many biomedical studies have focused on the role of MHC II in the initiation of autoimmune responses, since they can present both exo- and endogenous peptides to CD4^+^ T cells under pathological conditions. Recently, there have been reports of a lot of association examples between certain MHC II alleles and the risk of developing autoimmune diseases (ADs) (*Table*). This fact is one of the main reasons behind the development of an autoimmune process and explains the phenomenon of "autoimmunity" at the molecular level.

**Figure:**
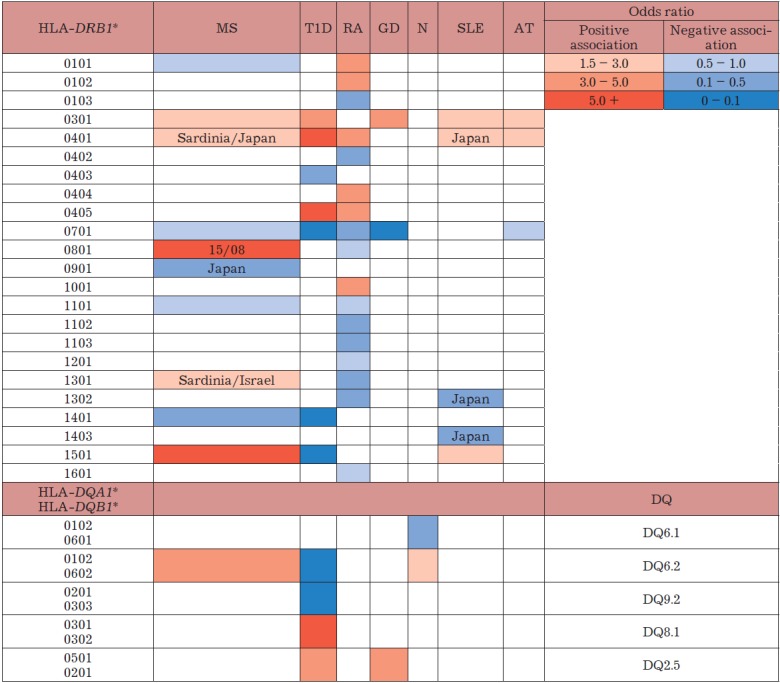
The association between the HLA-DRB1, HLA-DQA1, and HLA-DQB1 alleles and autoimmune diseases

MS – multiple sclerosis; T1D – type 1 diabetes; RA – rheumatoid arthritis; GD – Graves’ disease; N – narcolepsy, SLE – systemic lupus erythematosus; AT – autoimmune thyroiditis.

In patients with autoimmune diseases (such as multiple sclerosis (MS), systemic lupus erythematosus (SLE), type 1 diabetes (T1D), rheumatoid arthritis (RA), Graves’ disease (GD), etc.), auto-antibodies are synthesized and lymphocytes often penetrate into the target organ, leading to inflammation and partial destruction. These diseases are mostly chronic. Although the AD patienthood can often be stabilized for prolonged time periods, researchers still need to gain a detailed understanding of the disease mechanisms in order to develop an effective treatment strategy. Antigen presentation and further T cells activation are considered key components of the immune response in many diseases [7] and are often the therapy targets. Therefore, studying the peculiarities of antigen presentation (and the structure and features of MHC I and II proteins in particular) is of utmost importance.

Interestingly, the autoimmune diseases accompanied by autoantibody production are typically associated with MHC II, while the diseases not accompanied by this phenomenon are more commonly associated with certain MHC I alleles [8]. An association with haplotypes including gene clusters is also observed in many diseases, which is likely to be the result of a linkage disequilibrium of these genes that occurs during inheritance. Thus, many diseases are known to be associated with the DR3-DQ2 MHC II haplotype and with the MHC I HLA-B8 and HLA-A1 alleles, which are components of the extensive, conserved haplotype [8].

## THE POTENTIAL MECHANISMS OF A LINK BETWEEN MHC II ALLELES AND THE RISK OF DEVELOPING CERTAIN ADS

MHC II molecules are involved in the presentation of antigens, including autoantigens (*Fig. 1*). The immune response develops after the 13–18 amino-acid antigenic peptide is presented by APCs using a MHC II molecule and recognized by the respective T-cell receptor on the CD4^+^ T-cell surface. Dendritic cells, B cells, and macrophages can act as APCs [9].

MHC II is synthesized in the endoplasmic reticulum and leaves this compartment as a complex with the invariant chain (IC) [10]. The IC catalyzes the release of MHC II from the endoplasmic reticulum and prevents its aggregation. The IC undergoes proteolysis in late endosomes, and only its small fragment (CLIP) remains bound to MHC II. It is most likely that CLIP impedes the interaction between MHC II and nonspecific peptides by blocking their access to the binding pockets [9].

In late endosomes, CLIP is exchanged for a MHC-specific antigenic peptide [11]. HLA-DM, a nonconventional MHC class II molecule [12], plays a crucial role in this process. This molecule is not polymorphic and cannot interact with antigenic peptides; however, its structure is similar to that of other MHC II molecules. HLA-DM catalyzes the binding of antigenic peptide to HLA-DR and substantially increases the rate of this reaction [13, 14]. Hence, HLA-DM contributes to the specific binding of MHC II to high-affinity peptides. The MHC II–peptide complexes are then delivered to the plasma membrane to present peptides to CD4^+^ T cells. The interaction with these cells determines whether the immune response is started or not. If the immune response is initiated, CD4^+^ T cells activate naïve B cells for a subsequent production of specific antibodies/ autoantibodies (for presentation of self-antigens) and contribute to the recruitment of macrophages to the immune response. Autoreactive CD4^+^ T cells against a number of self-antigens have been identified in patients with AT, GD, and MS [15].

Several hypotheses exist regarding the emergence of autoimmune diseases mediated by a number of MHC II alleles. Thus, a positive or negative association between autoimmune diseases and various HLA alleles can be determined through the structural features of the antigen-binding groove of the MHC molecule, which is encoded by a certain allele and is responsible for peptide binding (particularly, the arrangement of certain amino acids in definite positions within this groove, such as the positions 11, 71, and 74 in the MHC β-chain) [8, 16]. These MHCs, with point substitutions, vary in their efficiency of binding and presentation of self-peptides [17, 18]. The structural MHC elements indirectly related to the initiation of an autoimmune response can reside not only in the peptide-binding groove, but also in its proximity, within the area directly in contact with the T-cell receptor. The MHC polymorphism in this region may cause either binding of autoreactive effector T cells or weak selection of regulatory T cells [6].

The autoimmune response can also be initiated through molecular mimicry, when exogenous viral or bacterial peptides share a structural similarity with endogenous self-peptides. Certain MHC alleles structurally adapted for presentation of these exogenous infectious peptides can also present structurally similar self-peptides, followed by the initiation of an autoimmune response [19].

Some examples have been reported when medication (abacavir in the treatment of HIV [8, 20] or even low-molecular-weight compounds such as Be^2+^ [21]) could be bound to the peptide-binding groove of a specific MHC allele, thus changing the specificity of peptide presentation and enabling presentation of self-peptides.

In some cases (e.g., in patients with the celiac disease [22] or RA [23]), the antigen peptides undergo post-translational modification and are preferentially presented on risk alleles.

The data on a positive or negative link between a number of MHC alleles and the risk of developing ADs are of exceptional importance in enabling successful target immunotherapy using drugs targeted at the stage of MHC II antigen presentation.

## THE DIVERSITY OF MHC II ALLELES ASSOCIATED WITH THE RISK OF DEVELOPING THE MOST COMMON AUTOIMMUNE DISEASES

**Multiple sclerosis**

Multiple sclerosis is a chronic neurodegenerative disease of the central nervous system (CNS) which is diagnosed in 0.1% Europeans and North Americans [24]. The risk factors for MS include polygenic inherited predisposition and a number of external factors, such as some infectious diseases, diet, and various social and climatic factors [25].

The disease has been shown to be clearly associated with the carriage of various genetic variants of MHC II. The most significant association between an extensive DR15-DQ6 haplotype (HLA-DRB1*1501/HLA-DRB5*0101/HLA-DQA1*0102/ HLA-DQB1*0602) and MS has been revealed in Caucasians [26]. Since all these alleles are characterized by a substantial linkage disequilibrium, it remained unclear for a long time which allele contributes the most to the predisposition to MS. A study focused on the association between MHC II genes and MS in African-Americans has allowed researchers to achieve some progress toward solving this problem. Linkage disequilibrium is less pronounced in this population, and the HLA-DRB1*1501 allele is the one most typically associated with MS, thus indicating that this allele plays the most important role among the three alleles in the haplotype [27]. Today, the HLA-DRB1*1501 allele has been recognized as the major risk allele for MS in Caucasians; its association with the disease has been demonstrated in most of the populations analyzed [28]. 

Multiple sclerosis has conventionally been regarded as a disease that affects women more often than men, since the female-to-male ratio between patients with relapsing-remitting (or secondary progressive) MS is 2.5 : 1. Interestingly, Hensiek et al. [29] have reported that women are also more likely to carry the HLA-DRB1*15 allele.

In addition to DRB1*1501, the universal risk allele for MS, other variants of the *HLA*-*DRB1 *gene positively associated with MS have been reported for different populations (*Table*). An association between DRB1*03 and MS has been revealed in many European populations; the risk of developing MS is significantly higher in homozygous carriers [30]. In patients from Sardinia and Japan, the HLA-DRB1*04 allele cluster was found to be also positively associated with MS, in addition to HLA-DRB1*03 [31, 32]. The HLA-DRB1*13 allele, which was also detected within the HLA-DRB1*1303/ HLA-DQB1*0301 haplotype in MS patients from Sardinia (Italy), was also found to be associated with MS in Israelis [33]. A strong positive association between carriage of the HLA-DRB1*08 variant and the risk of developing MS was found to exist in Caucasians with the HLA-DRB1*15/08 genotype [34, 30].

The HLA-DRB1*14 variant was found to be a major protective allele (i.e., the allele negatively associated with the disease and that reduces its risk compared to the average population risk) in Northern Europeans [34]. The HLA-DRB1*01, *07, and *11 allele clusters are also regarded as protective, albeit to a lesser extent, in Caucasians [30, 35, 36]. The HLA-DRB1*11 variant also exhibits a pronounced protective effect in African-Americans [37] and residents of Sardinia [32]. The DRB1*0901 allele can be considered as protective against MS in Japanese natives; its frequency across Asian countries is normally higher than it is in other countries [31].

Recent studies have demonstrated that the effect of protective and risk alleles can mutually compensate in heterozygous carriers. Thus, the effect of the HLA-DRB1*15 and *03 risk alleles was found to be mitigated in the presence of the protective HLA-DRB1*14 or *11 variant [30, 34].

It should be mentioned that carriage of certain HLA alleles is associated with the age of onset in multiple sclerosis. Thus, carriage of HLA-DRB1*1501, the major risk allele for MS, is associated with earlier onset of the disease in Caucasians [38], while carriers of the DRB1*0405 allele display an earlier onset of the disease in the Japanese population [31]. It is known that in patients with MS, the immune system attacks the components of the myelin sheath formed by oligodendrocytes [39]. A number of autoantigens in MS have been identified: the myelin binding protein (MBP), proteolypid protein (PLP), myelin oligodendrocyte glycoprotein (MOG), and the myelin-associated glycoprotein (MAG). Today, MBP is considered to be the most important of these autoantigens. MBP-specific CD4^+^ T cells have been revealed in the brain and spinal cord tissues of MS patients [40], while APCs presenting the main encephalitogenic peptide MBP (a fragment consisting of amino acids 85–99) have been detected directly in demyelination foci [41, 42]. MHC II molecules encoded by HLA-DRB1*1501, the universal risk allele for MS that binds to the MBP85-99 fragment, play a crucial role in the presentation of this peptide on the surface of APCs. An autoimmune response to this complex in humanized mice has been reported [43], which can be regarded as the main mechanism explaining the observed association.

**Type 1 diabetes**

Type 1 diabetes (T1D), found in 0.06–0.15% of the population, is caused by an autoimmune inflammation of pancreatic tissue, resulting in impaired insulin secretion [41, 44]. It has been demonstrated that autoreactive T cells are derived from normal cells in patients with T1D, due to the presentation of insulin fragments on MHC II molecules. The association between T1D and the DRB1*03 and DRB1*04 allele clusters was described earlier [6, 45]. Later, an association between this disease and *DQB1 *variants was revealed; the alleles of this gene (e.g., HLA-DQB1*0302 (DQ8) or HLA-DQB1*0201 (DQ2)) are associated with a high risk of T1D only when encoding a neutral amino acid (e.g., Ala) at position 57. If this position is occupied by the negatively charged aspartic acid as is the case for the DQB1*0602 (DQ6.2) and DQB1*0303 (DQ9) alleles [46], the respective allele will exhibit a protective activity [6, 47]. It has been shown that the amino acid residue 57 is located in the P9 pocket of the peptide-binding groove and is responsible for the formation of the DQA1– DQB1 heterodimer [47]. Probably, if aspartic acid is substituted for a neutral amino acid at this position, the specificity of the MHC molecule will be changed and it will become able to present insulin fragments. Interestingly, the HLA-DRB1*0301, HLA-DRB1*0405, and HLA-DRB1*0401 alleles are positively associated with T1D, while the very similar HLA-DRB1*0403 allele is negatively associated with T1D [46, 48]. It is possible that in patients with this disease, the antigen-binding grooves in the positively and negatively associated MHC II molecules are structurally similar and have only a single-point mutation affecting the specificity of peptide binding. Furthermore, the HLA-DRB1*0701, HLA-DRB1*1401, and HLA-DRB1*1501 alleles also exhibit a strong protective activity [46].

**Rheumatoid arthritis**

Rheumatoid arthritis is a chronic inflammatory disease that affects the joints. Almost all RA patients carry the HLA-DRB1*0401, HLA-DRB1*0404, HLA-DRB1*0405 or HLA-DRB1*0101 allele [49-51]. Interestingly, the β chains of MHC II are products of these alleles and share an amino acid motif inside the peptide-binding groove at positions 67–74, which forms the so-called "degenerate epitope" [41, 18]. It has been demonstrated that the point mutations within the degenerate epitope change the charge, affect the association with RA, and are often the only differences between the risk and protective alleles DRB1*0103, DRB1*0402, DRB1*0701, DRB1*1102, and DRB1*1301 [6, 7, 49, 52]. A positive association between *DQB1 *variants and RA has also been demonstrated [53], although this association is probably caused by a linkage disequilibrium with *DRB1 *alleles [54].

**Graves’ disease**

Graves’ disease (GD), also known as toxic diffuse goiter or Basedow’s disease, is an autoimmune disorder caused by excessive secretion of thyroid hormones by the diffuse tissue of the thyroid gland, resulting in thyroid hormone poisoning (thyrotoxicosis). This disease is eight times more likely to affect women than men. It typically develops in middle-aged adults (usually between 30 and 50 years of age). The observed significant familial predisposition to GD indicates that the genetic component substantially contributes to the development of this disease. It has been demonstrated that predisposition to both GD and RA is associated with the degenerate motif in the *DRB1 *gene product (namely, with the amino acid at position 74 in the β chain of MHC II). Thus, the MHC molecule encoded by the GD-associated DRB1*03 variant and the product of the protective variant DRB1*07 carry Arg and Glu, respectively, at position 74 [55, 56]. It is worth mentioning that the protective MHC II alleles and the risk alleles also differ for T1D and RA in terms of the amino acid residing at position 74 [6]. The position 74 in the β chain of MHC might be exceptionally important, since this amino acid residue is located within the P4 pocket, where the peptide-binding motif of MHC overlaps with the T-cell receptor docking site [57].

**Narcolepsy**

Narcolepsy is a chronic neurodegenerative disease characterized by excessive daytime sleepiness and disturbed nighttime sleep [41, 58]. It is a complex disease whose etiology has yet to be fully elucidated. Its presumably autoimmune nature has been attributed to an explicit association with the DQB1*0602 MHC II allele, as almost 100% of patients diagnosed with narcolepsy carry this allele [59]. Findings of an autoimmune T-cell response in patients with this disease have also been reported [60]. Since the structurally very similar HLA-DQB1*06011 allele (differing from the HLA-DQB1*0602 by only 9 codons in the β-chain gene) is protective for this disease [61], it is likely that the association/protection mechanism is also related to variations in the binding strength of the presented peptide and T-cell receptor docking in this case. An antigen whose fragments can be presented by the HLA-DQ6.2 product (DQA1*0102/DQB1*0602) has not yet been conclusively identified; however, a hypothesis has been put forward that this antigen could be orexin (hypocretin), a neurotransmitter that is involved in sleep regulation and synthesized in the hypothalamus [51]. The crystal structure of an HLA-DQ6.2 molecule bound to the peptide (a hypocretin derivative) has been deciphered [62].

Interestingly, the accumulation of data on a link between MHC II and a risk of developing ADs has revealed that the same variants are associated with several other diseases. These variants are often found in extensive haplotypes that involve the *DRB1, DQA1*, and *DQB1 *genes and are inherited together due to a strong linkage disequilibrium. The DR3-DQ2 and DR4- DQ8 alleles within the so-called extended haplotypes (DRB1*03/DQA1*0501/DQB1*0201 and DRB1*04/ DQA1*0301/DQB1*0302, respectively) are associated with T1D [41, 63]. Meanwhile, DR3 is also associated with MS, GD, SLE, and AT; therefore, it is referred to as the "autoimmune haplotype" [6]. DR4 is also associated with a number of diseases, including RA and AT. On the other hand, it is worth mentioning that the HLA-DRB1*0701 allele exhibits a protective effect in many diseases, such as MS, T1D, RA, GD, and AT (*Table*).

Recent studies have demonstrated that the extent of any association between a certain MHC allele and autoimmune diseases is also dependent on the regulated level of expression of such an allele. Furthermore, it has been revealed that increased expression of a particular MHC II allele may change the T-cell receptor repertoire during T-cell maturation in the thymus gland and affect the survival and expansion of mature T-cell clones. It has been shown that the MHC expression can be regulated at both the transriptional and post-transcriptional levels [64].

## CONCLUSIONS

Most autoimmune diseases are caused by a number of factors (including genetic, social, and climatic ones) and depend on a patient’s age and sex, smoking, past history of infections, etc. However, the risk of developing an AD significantly increases in patients with genetic predisposition, which is often dependent on carriage of certain MHC II genes. The MHC II variants whose carriage may lead to the development of an autoimmune disease in a particular person have been characterized. A number of MHC II alleles exhibiting protective activity against specific diseases have been reported. A cluster of MHC II genes, either positively or negatively associated with the diseases, can vary depending on a person’s ethnicity. More and more structural data on autoantigen presentation on MHC II molecules is becoming available each year. Information on the structures of several trimolecular MHC II–peptide–T-cell receptor complexes has been obtained. An integrated approach is needed for a comprehensive understanding of the mechanisms of AD induction and for developing novel therapeutic modalities. Such an approach should include an in-depth investigation of the elemental stages of MHC II antigens presentation mechanism, the basis of the protective activity exhibited by different MHC II alleles, the different characteristics of MHC II autoantigen loading, including the kinetic peculiarities, and the eliciting of a further autoimmune response involving activated CD4^+^ T cells. 

## Abbreviations

AD: autoimmune disease
MHC: major histocompatibility complex
HLA: human leukocyte antigen
APC: antigen-presenting cells
MS: multiple sclerosis
SLE: systemic lupus erythematosus
T1D: autoimmune type 1 diabetes
RA: rheumatoid arthritis
GD: Graves’ disease
N: narcolepsy
AT: autoimmune thyroiditis
LD: linkage disequilibrium
IC: invariant chain
